# Receptors underlying an odorant's valence across concentrations in *Drosophila* larvae

**DOI:** 10.1242/jeb.247215

**Published:** 2024-05-14

**Authors:** Sarah Perry, Jonathan T. Clark, Paulina Ngo, Anandasankar Ray

**Affiliations:** ^1^ Graduate program in Genetics, Genomics, and Bioinformatics, University of California, Riverside, Riverside, CA 92521, USA; ^2^ Interdepartmental Neuroscience Program, University of California, Riverside, Riverside, CA 92521, USA; ^3^ Department of Molecular Cell and Systems Biology, University of California, Riverside, Riverside, CA 92521, USA; ^4^ Center for Disease Vector Research, University of California, Riverside, Riverside, CA 92521, USA

**Keywords:** Odorant receptor, Aversion, *Drosophila*, Olfaction

## Abstract

Odorants interact with receptors expressed in specialized olfactory neurons, and neurons of the same class send their axons to distinct glomeruli in the brain. The stereotypic spatial glomerular activity map generates recognition and the behavioral response for the odorant. The valence of an odorant changes with concentration, typically becoming aversive at higher concentrations. Interestingly, in *Drosophila* larvae, the odorant (E)-2-hexenal is aversive at low concentrations and attractive at higher concentrations. We investigated the molecular and neural basis of this phenomenon, focusing on how activities of different olfactory neurons conveying opposing effects dictate behaviors. We identified the repellant neuron in the larvae as one expressing the olfactory receptor Or7a, whose activation alone at low concentrations of (E)-2-hexenal elicits an avoidance response in an Or7a-dependent manner. We demonstrate that avoidance can be overcome at higher concentrations by activation of additional neurons that are known to be attractive, most notably odorants that are known activators of Or42a and Or85c. These findings suggest that in the larval stage, the attraction-conveying neurons can overcome the aversion-conveying channels for (E)-2-hexenal.

## Teaser

**Summary:** An odorant that activates only one odorant receptor, Or7a, in *Drosophila* larvae becomes strongly attractive at higher concentrations, by recruiting activation of additional odorant receptors.

## INTRODUCTION

In most animals, the olfactory system is responsible for detecting volatile chemical cues in the environment and conveying that information to the brain so that behavior such as attractiveness or repellency can be determined. In both vertebrates and flies, primary olfactory receptor neurons (ORNs) are highly specialized cells that typically express single or a few receptor proteins (Couto et al., 2005; Fishilevich and Vosshall, 2005). ORNs expressing the same receptor project their axons to the same glomeruli in the brain. In *Drosophila*, the receptor-to-glomerulus map is highly stereotypical and has been extensively studied. A given odorant may activate several receptor types and therefore several ORN classes to produce a distinct pattern of activation across the glomeruli of the antennal lobe (Wang et al., 2003).

The larval olfactory system represents a simplified version of the adult system. Rather than over 1000 ORNs belonging to ∼50 classes, the larvae have 21 ORNs on each side of the head, with their dendrites housed in the dome sensillum. Each of these 21 sensory neurons is thought to belong to a different class and express a different receptor, except for two neurons that co-express receptor pairs (Or33b/Or47a and Or94a/Or94b) (Fishilevich et al., 2005; Kreher et al., 2005). Typically, a ‘tuning’ *Or* is singularly expressed along with the obligate co-receptor *Orco* (Larsson et al., 2004). As in adults, different ORNs project their axons to different glomeruli in the larval antennal lobe (Fishilevich et al., 2005; Kreher et al., 2005).

Most odorants naturally activate more than one ORN channel (Hallem and Carlson, 2006; Hallem et al., 2004; Kreher et al., 2008; Kreher et al., 2005; Montague et al., 2011, 2009); however, in *Drosophila* it is known that activation of a single glomerulus via a single ORN class is sufficient to drive aversion or attraction behavior, suggesting that each ORN is associated with a particular behavior (Bellmann et al., 2010; Semmelhack and Wang, 2009; Suh et al., 2004; Stensmyr et al., 2012). For example, adult *Drosophila* find low concentrations of apple cider vinegar (ACV) attractive via activation of DM1 and VA2 glomeruli via Or42a and Or92a, respectively. As concentrations of ACV increase, additional glomeruli are recruited, including the repellant channel governed by DM5 via Or85a. Activation of the Or85a channel is sufficient to override Or42a-mediated attraction and cause higher ACV concentrations to become repellant (Semmelhack and Wang, 2009). In this case, a low concentration of an odorant activates a few receptors and is perceived as attractive. As odor concentration increases, additional receptors are recruited and the behavioral valence of an odorant switches from attractive to aversive.

In *Drosophila* larvae, most odorants tested so far have been found to be attractive, although aversive responses have also been noted in a small fraction of odorants (Kreher et al., 2008; Cobb et al., 1997). The use of optogenetic techniques has demonstrated that activation of a single ORN type can effectively drive chemotaxis behavior. While many larval ORNs direct attractive behavior, only three (Or33b, Or45a and Or49b) are known to mediate repellency (Bellmann et al., 2010; Launikonis et al., 2011). While a powerful approach, optogenetics relies on the availability of strong *Or* promoter-Gal4 drivers, which are not available for all larval receptors (Couto et al., 2005; Fishilevich and Vosshall, 2005; Fishilevich et al., 2005; Kreher et al., 2005). Because of this, the behaviors associated with all larval Ors and the neurons they are expressed in are yet to be determined.

To investigate the role of individual ORNs in larval olfactory behavior, we used an alternative approach involving very low concentrations of odorants to selectively activate single receptors (Mathew et al., 2013) and observed the elicited behavior. We identified a repellant effect with Or7a, which is singularly activated by low concentrations of (E)-2-hexenal. Interestingly, as (E)-2-hexenal increases in concentration, it becomes highly attractive, potentially as a result of the activation of additional attractive receptors, which are able to overcome Or7a-mediated repellency. Our findings suggest that the interaction between attractive and repellant pathways in the case of this one odorant in the larval stage may be the opposite of what has been observed previously in the adult with other odorants (Semmelhack and Wang, 2009).

## MATERIALS AND METHODS

### Larval behavioral assay

The larval odor preference assays were performed as previously reported with some modifications (Kreher et al., 2008). Here, 25 µl of the odor solution or solvent was presented in inverted 0.2 ml PCR tube caps.

### Fly lines used

Unless otherwise indicated, wCS (*w-Canton-S*) was used as the wild-type control line in this study. The *Elav-Gal4/Cyo; UAS-DCR2* stock used for RNAi experiments was made from Bloomington stocks 8765 and 24,651. The *Or7a-RNAi* stock is Vienna Drosophila Resource Center (VDRC) v107874. CRISPR mutations were created as follows. Targeting oligos (CTTCGTGGTCGGTCGACTGCCATC, AAACGATGGCAGTCGACCGACCAC) were designed using the online tool available at https://flycrispr.org/. These oligos were cloned into BbsI-digested pU6-BbsI-chiRNA (Addgene plasmid #45946). The U6-chiRNA cassette was then isolated using EcoRI and KpnI digestion and cloned into pattB. We then used the Phi-C31 system to integrate pattB-U6-chiRNA into the attP40 docking site. *attP40[w^+^,* *pU6-Or7a-chiRNA]/Cyo* individuals were then mated to *vas-Cas9* flies (BL# 51324). *attP40[w^+^,* *pU6-Or7a-chiRNA]/+; vas-Cas9/+* daughters were then crossed to *FM7* balancer males to capture any *Or7a* CRISPR mutations. Eighteen isogenic lines were created from w^−^ progeny and the pertinent segment of the *Or7a* locus was PCR amplified and sequenced [primers: CACATCATCGCTAGCTT GGT (also used for sequencing reaction), ACTCGTGTGGCCACAAAGCA] from an individual from each line to look for indels at the CRISPR target site. The lines *Or7a-3* and *Or7a-7* may still be segregating vas-Cas9. The mutant *Orco^−/−^* (previously called *Or83b^1^*) was obtained from Bloomington stock center.

### Electrophysiology

Electrophysiology recordings were performed as previously reported (Kreher et al., 2008) using 3–7 day old flies of the indicated genotypes. Diagnostic odorants were used to distinguish individual classes of ORNs in adult fly sensilla (ab1–ab7) and therefore unequivocally identify the target ORN for testing (Hallem and Carlson, 2006; Hallem et al., 2004). Odor stimulus flow was 12 ml s^−1^. The tested odorants were dissolved in paraffin oil at their respective concentrations (e.g. 10^−4^ dilution) and 50 μl of the odor solution was applied to cotton in a Pasteur pipette cartridge. The headspace from the cartridge was puffed into the carrier flow of humidified air and directed over an insect’s head. Larval dome sensillum recordings were performed similarly to the report by Kreher et al. (2005), with the exception that amplifier settings were optimized to capture action potentials rather than membrane potentials. The 10 s recordings of each trace covered 1 s baseline, followed by 1 s of odor stimulation, and 8 s of post-stimulus activity. The response to a stimulus was reported as a spikes per second value based on the counts during the 1 s stimulus minus the counts during the 1 s spontaneous activity prior to the stimulus.

## RESULTS

### (E)-2-Hexenal avoidance changes to attraction at higher concentrations

**Fig. 1.**
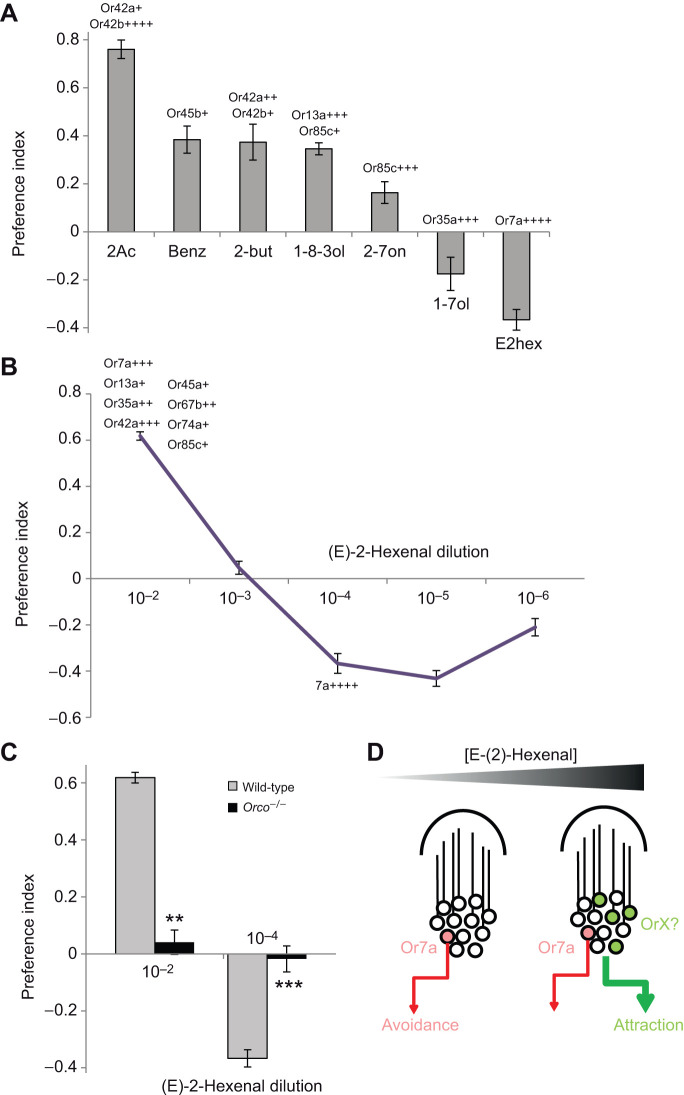
**Low concentrations of E(2)-hexenal are repellant to larvae via an Or-dependent circuit.** (A) Larval preference indices in a two-choice plate assay to lower concentrations (10^−4^ dilution) of an odor panel selected to activate single or a few receptors. Known receptor activities to the odors are represented above the data points (level indicated by +). (B) Dose–responses of larvae to (E)-2-hexenal at different dilutions. Known receptor activities to particular concentrations are represented above the data points (*N*=5–15). (C) Preference indices of wild-type (wCS) and Orco^−^ mutant larvae to selected concentrations of (E)-2-hexenal (*N*=6–10). Error bars represent ±s.e.m. (2-tailed Mann–Whitney *U*-test: **P≤0.01 ***P≤0.001). (D) Model explaining the larval response to low or high concentrations of (E)-2-hexenal. OrX represents one or more unknown receptors. 2Ac, ethyl acetate; Benz, benzaldehyde; 2-but, 2-butanone; 1-8-3ol, 1-octen-3-ol; 2-7on, 2-heptanone; 1-7ol, 1-heptanol; E2hex, (E)-2-hexenal.

Much olfactory behavioral work in larvae has been done with relatively high concentrations of odorants. The disadvantage of this approach is that multiple receptor classes are activated by each odorant and it is difficult to determine the contribution of each channel. However, many of the same odorants at lower concentrations (10^−4^ dilution) activate only one or two receptor types (Mathew et al., 2013; Kreher et al., 2008). Based on the known activation profiles, we tested the larval chemotaxis response to a panel of odorants at low concentrations in order to determine the role of single ORNs (Fig. 1A). Consistent with what is known thus far about larval olfaction, most odors elicited an attractive or neutral response. However, one odorant, (E)-2-hexenal, caused a robust avoidance response. Previously published data demonstrate that low concentrations of this odorant only activated one receptor, Or7a, in the larvae (Kreher et al., 2005, 2008), suggesting that Or7a could be a repellant pathway.

In order to test whether a stronger dose of the odorant led to greater repellency, we performed a dose–response analysis. Interestingly, as (E)-2-hexenal increases in concentration, it becomes very attractive (Fig. 1B). Previously published electrophysiology data indicate that at least seven receptors in addition to Or7a are activated at the higher attractive concentration (Kreher et al., 2008).

Although Or7a appears to be a promising candidate for (E)-2-hexenal repellency, larvae could also putatively detect odors via ionotropic (Ir) or gustatory receptors (Gr). To determine whether the behavioral effect was mediated by Or receptors, we tested responses to 10^−2^ and 10^−4^ dilutions of (E)-2-hexenal in *Orco^−/−^* mutant larvae. Both the respective attraction and repulsion were lost, indicating that these behaviors are indeed Or mediated (Fig. 1C).

### Or7a neuron activation causes avoidance of low concentrations of (E)-2-hexenal

In order to test the role of Or7a in the aversion response to low concentrations of (E)-2-hexenal, we performed RNAi knockdown using the pan-neuronal *Elav-Gal4* driver and a *UAS-RNAi Or7a* line from VDRC. This knockdown caused a significant reduction, but not a complete loss, of (E)-2-hexenal repellency (Fig. S1A).

**Fig. 2.**
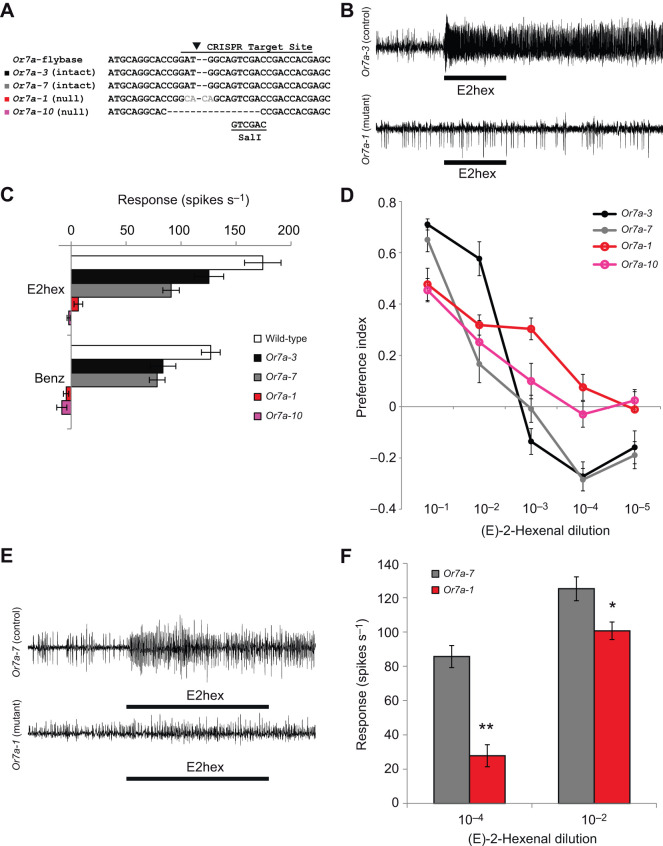
**Behavioral and physiological responses to low concentrations of E-(2)-hexenal are *Or7a* dependent.** (A) Sequences of selected *Or7a* alleles generated by CRISPR surrounding the predicted Cas9 cut site. Lines *Or7a-3* and *Or7a-7* showed a sequence identical to the wild-type sequence from FlyBase. Lines *Or7a-1* and *Or7a-10* exhibited indels causing frame-shift mutations and are assumed to be null alleles. (B) Representative traces from single sensillar electrophysiological recordings from ab4 sensilla in *Or7a-3* control and *Or7a-1* mutant lines to (E)-2-henenal at 10^−4^ dilution. (C) Electrophysiology responses from ab4 sensillum (A and B neurons) in response to Or7a ligands [10^−4^ dilution (E)-2-hexenal, 10^−2^ dilution benzaldehyde] from wild-type, control (*Or7a-3*, *Or7a-7*) and mutant (*Or7a-1*, *OR7a-10*) lines (*N*=4–9; means±s.e.m.). Statistical analysis was performed using a two-tailed Student's *t*-test. *P*-values can be found in Table S1. (D) Mean preference index of dose–responses of larvae to (E)-2-hexenal for *Or7a-1* and *Or7a-10* (predicted null) and *Or7a-3* and *Or7a-7* (control) lines (*N*=8–12; means±s.e.m.). Statistical analysis was performed using a 2-tailed Mann–Whitney *U*-test. All *P*-values can be found in Table S2. (E) Representative traces from larval dome sensillum electrophysiological recordings in control (*Or7a-7*) and mutant (*Or7a-1*) animals in response to (E)-2-hexenal at 10^−4^ dilution. (F) Mean of dome sensillum responses to (E)-2-hexenal at 10^−2^ and 10^−4^ dilution in control (*Or7a-7*) and mutant (*Or7a-1*) animals (*N*=6–7; means±s.e.m.). Unpaired *t*-test: *P<0.05, **P<0.0001.

Additionally, we created an *Or7a* knockout using the CRISPR system. We recovered 18 putative mutant lines that were isolated and sequenced. Sequencing showed 15 of these 18 lines had indels at the CRISPR cut site, all of which were predicted to result in frame-shift mutations causing non-functional protein products. Three lines had an ‘intact’ *Or7a* sequence identical to the genomic sequence available from FlyBase and could therefore be used as controls. Two intact (*Or7a-3* and *Or7a-7*) and two putatively null lines (*Or7a-1* and *Or7a-10*) were selected for further testing (Fig. 2A).

While it is possible to record neuronal activity from the larval dome sensillum, it is impossible to sort the action potential responses of a single ORN using this technique. An alternative approach is to use a genetic approach with larvae of the Or co-receptor mutant *Orco^−/−^* where only one class of neuron is genetically rescued to be functional using a specific Or-Gal4 driver line to express UAS-Orco (Grillet et al., 2016). However, a faithfully expressing Or7a-Gal4 driver line has not been successfully created yet, although Or7a mRNA is clearly demonstrated with *in situ* hybridization (Fishilevich et al., 2005).

*Or7a* is also expressed in the adult antennal neuron type called ab4A (Couto et al., 2005; Hallem and Carlson, 2006; Hallem et al., 2004). We recorded from these sensilla in the *Or7a* mutants to validate that the receptor is non-functional in our putative null lines. Indeed, while the intact lines *Or7a-3* and *Or7a-7* did respond to the known ligands (E)-2-hexenal and benzaldehyde, mutant lines *Or7a-1* and *Or7a-10* did not (Fig. 2B,C). The neighboring ab4B neuron expressing *Or56a* retained responses to geosmin in both the mutant and control lines (Fig. S1B) (Stensmyr et al., 2012). We also examined responses to (E)-2-hexenal in the larval dome sensillum. Responses to (E)-2-hexenal at 10^−4^ dilution were substantially reduced in the mutant compared with the control line and a reduction in response was even observed at 10^−2^ concentration (Fig. 2E,F).

We next examined larval chemotaxis towards (E)-2-hexenal in the mutant and control lines. Avoidance of (E)-2-hexenal at low concentrations was completely abolished in the mutant lines, but all lines maintained attraction to higher concentrations (Fig. 2D). These results demonstrate that *Or7a* is required for avoidance of low concentrations of the odorant.

### Odorants that activate Or42a and other Ors override avoidance of E-2-hexenal

**Fig. 3.**
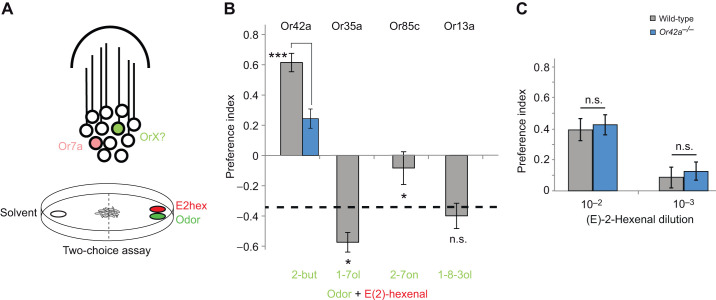
**Or7a neuron driven repellency can be overridden by activity in other olfactory neurons.** (A) Schematic diagram showing a larval chemotaxis assay for activating Or7a and an additional receptor neuron(s) (represented by OrX). (B) Larval preference indices to odors (10^−4^ dilution) in addition to (E)-2-hexenal (10^−4^ dilution). Known receptors activated by the odors are represented above the bars. The response to (E)-2-hexenal alone at the same concentration is shown with a dashed line (*N*=6–10; means±s.e.m.). Pairwise comparisons between odor plus (E)-2-hexenal and to (E)-2-hexenal alone were performed using two-tailed Student's *t*-test: *P<0.05, ***P≤0.001 (n.s., not significant). (C) Larval preference indices to (E)-2-hexenal at 10^−2^ and 10^−4^ dilution in wild-type and *Or42a* mutant flies (*N*=10; means±s.e.m.). Two-tailed Student's *t*-test.

Previously published electrophysiology data show that in addition to Or7a, at least seven other receptors are activated at the attractive concentration of (E)-2-hexenal (10^−2^ dilution) (Kreher et al., 2008): Or42a, Or35a, Or67b, Or85c, Or74a, Or45a and Or13a, with the strongest activation in Or42a and Or35a. A possibility is that one or more of these neurons expressing receptors conveys the attractive behavior and is able to override Or7a neuron-mediated repellency as proposed in one model. In order to test whether these Or channels are able to overcome Or7a driven repellency, we presented the larvae with aversive (E)-2-hexenal (10^−4^ dilution) in combination with another odorant selected such that they activate the strongest responding receptors for overcoming the aversion (Fig. 3A) (Kreher et al., 2008). A few of the odorants were indeed able to reduce the aversion behavior, and in some cases cause attraction. Interestingly, while the Or42a-activating ligand 2-butanol (10^−4^ dilution) was able to overcome the repellency and cause attraction in an Or42a-dependent manner (Fig. 3B), 1-heptanol (10^−4^ dilution), an Or35a-activating ligand, did not reduce aversion to (E)-2-hexenal (10^−4^ dilution) (Fig. 3B). When the aversive concentration of (E)-2-hexenal was tested alongside 2-heptanone (10^−4^ dilution), an activator of the moderately active receptor candidate Or85c, there was a moderate decrease in aversion. However, 1-octen-3-ol, an Or13c activator, had no significant effect (Fig. 3B). If indeed this is due to dominance of the attractive channels Or42a and Or85c over the aversive Or7a channel, it would suggest that activity of an aversive channel is sufficient to override the attractive response (Semmelhack and Wang, 2009).

While multiple attractive Or channels could be involved in the attraction to the higher concentrations of (E)-2-hexenal in combination, which would be difficult to test, we picked one receptor that showed greatest activity. In order to test whether activity of Or42a in the neurons is necessary for the valence reversal, we presented *Or42a^−/−^* mutant larvae (Kreher et al., 2008) with (E)-2-hexenal alone at 10^−2^ and 10^−3^ dilution, levels which are attractive to wild-type larvae. If Or42a alone were sufficient for the attractive response to high concentrations of (E)-2-hexenal, then we would expect the attractive response to be reduced in these mutant larvae. However, we found that there was no significant difference in the attractive preference indices of wild-type and *Or42a^−/−^* mutant larvae at the 10^−2^ and 10^−3^ dilutions (Fig. 3C), indicating that although activation of the Or42a neuron is sufficient to induce attraction for other odorants present in a mixture, it is not necessary and its absence is likely compensated for by neurons expressing one or more of the other receptors that detect (E)-2-hexenal.

## DISCUSSION

Here, we demonstrate a few novel findings in insect olfaction. First, we identified a previously unreported repellant channel in *Drosophila* larvae mediated by Or7a. This receptor elicits avoidance behavior when activated by low concentrations of (E)-2-hexenal. Higher concentrations of the same odorant recruit additional receptors and the Or7a avoidance response is overridden to produce an attractive response. We further demonstrate that activation of attractive ORNs is able to completely reverse Or7a-driven repellency. *Or7a* is expressed in both the larval and adult stages of *Drosophila melanogaster* as demonstrated with RNA *in situ* hybridization studies (Fishilevich et al., 2005). In the adult, *Or7a* has recently been implicated in conveying repellency towards spatial repellents such as pyrethroids (Wang et al., 2021), suggesting that the aversive behavior associated with the receptor-expressing neuron is similar across life stages.

Interestingly, another class of larval neurons that causes aversion expresses Or49a, and detects iridomyrmecin from parasitoid wasps (Launikonis et al., 2011) and is inhibited by butanol (Hoare et al., 2008). Another role for Or7a in the adult was thought to be detection of a conspecific male-produced pheromone 9-tricosene (Lin et al., 2015), although (E)-2-hexenal contamination could be contributing to the response (Schorkopf et al., 2018 preprint). (E)-2-Hexenal is known to be a green leaf volatile that is released from plants that are damaged by herbivorous insects, and Or7a could play a role in the detection of this in flies (Tumlinson et al., 2019; Matsui and Koeduka, 2016; Heil, 2014). These findings suggest that Or7a may be imparting aversion in both the adult and larval stages.

Different models can be invoked to explain this phenomenon. The lower concentration of (E)-2-hexenal activates Or7a, which conveys repellency, but higher concentrations are attractive through activation of other Ors, which in turn override aversion. High concentrations of (E)-2-hexenal are detected by several receptors (Kreher et al., 2008). The exact mechanism underlying this valence switch is not yet clear because the contribution of each of these receptors to the attractive response is currently unknown. However, there are at least two possibilities. First, one or a combination of these other receptors may be able to directly override the Or7a channel such that despite functional activation of the latter, larvae are attracted. The other possibility is that at such high concentrations, the Or7a neuron becomes saturated and loses its ability to affect behavior, at which point the other, attractive channels emerge as the primary drivers of olfactory behavior. A similar effect has been demonstrated in the CO_2_-detecting neurons of mosquitoes (Turner et al., 2011) and in larval stages as well (Boyle et al., 2016). This model is identical to the first in some respects, but additionally the higher concentrations of (E)-2-hexenal cause prolonged saturating responses from the aversive Or7a neurons in a manner that blocks the ability to detect the odor gradient as it pertains to aversion. However, we cannot determine the relative contribution of the second model to the behavioral change as it is extremely difficult to record just the activity of Or7a neurons or the connected second-order projection neurons in the larval stage. It is also possible that the two odorant plumes interact and elicit non-linear responses from the various Ors. We hope future studies will be able to analyze the contributions of the different possibilities.

In adult flies, it has been shown that activation of a repellant channel in addition to attractive channels results in repulsion, suggesting that repellant glomeruli can override attractive ones. However, what we observed here suggests that the opposite is true in the larval behavior towards (E)-2-hexenal – the attractive channels overcome the repellant counterpart. This is also supported by a previous study showing that activating all Or-expressing neurons at once using channel rhodopsin results in attraction (Bellmann et al., 2010). This may be advantageous to larvae because they spend much of their time in a high-odor environment where their only navigational tasks involve short-range expeditions to locate and remain near rich food sources. Larvae are also likely to encounter odor gradients more than the odor plumes which adult flies experience during flight. The interplay between the larval attractive and repellant channels may allow the animal to respond to these kinds of stimuli. Given their lifestyle, what purpose do repellant channels serve in larvae? In the case of Or7a, whose known ligands are (E)-2-hexenal, benzaldehyde and related compounds, this channel may serve to help the animal evaluate and modulate responses to food sources.

## Supplementary Material

10.1242/jexbio.247215_sup1 Supplementary information
