# Supplementary material for: Receptors underlying an odorant's valence across concentrations in Drosophila larvae
Source: J Exp Biol. 2024 May 14;227(9):jeb247215. doi: 10.1242/jeb.247215 (PMC11166451; doi:10.1242/jeb.247215)
Supplement: Supplementary information [file jexbio-227-247215-s1.pdf]

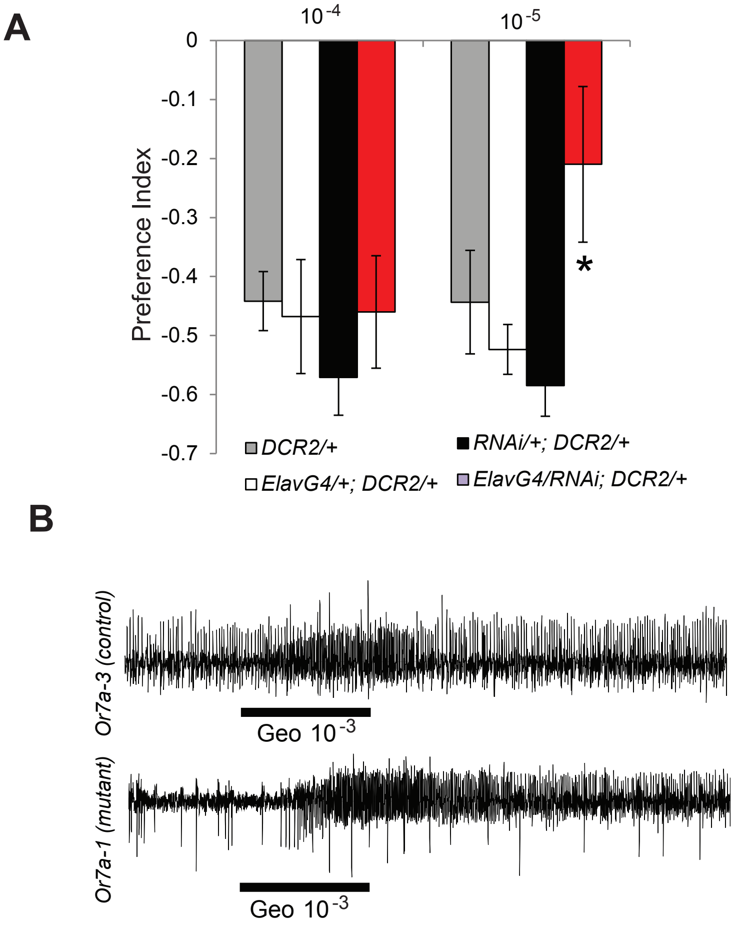

Fig. S1. Or7a Supplementary information. A) Larval preference indices of Or7a-RNAi knock-down animals and selected control genotypes to lower concentrations of E(2)-hexenal. (N = 4-10) **B)** Example electrophysiological responses of the ab4B neuron to geosmin in control and Or7a mutants.

Journal of Experimental Biology • Supplementary information

Table S1. Statistical analysis related to Fig. 2C

		Wild-type	Or7a-3	Or7a-7	Or7a-1
E(2)-hexenal 10^{-4}	Or7a-3	0.036473*			
	Or7a-7	0.000361***	0.047842*		
	Or7a-1	4.38E-07****	2.75E-06****	3.18E-07****	
	Or7a-10	2.63E-05****	7.2E-05****	6.89E-06****	0.135852$^{n.s.}$
Benzaldehyde 10^{-2}	Or7a-3	0.012388*			
	Or7a-7	0.000695***	0.710639$^{n.s.}$		
	Or7a-1	4.1E-09****	1.21E-05****	1.25E-07****	
	Or7a-10	1.26E-06****	0.000338***	1.19E-05****	0.419795$^{n.s.}$

Student's *t*-test, 2-tailed.

Table S2. Statistical analysis related to Fig. 2D

		Or7a-3	Or7a-7	Or7a-1
E(2)-hexenal 10^{-1}	Or7a-7	0.2713$^{n.s.}$		
	Or7a-1	0.0058**	0.0588$^{n.s.}$	
	Or7a-10	0.0009***	0.0173*	0.7949$^{n.s.}$
E(2)-hexenal 10^{-2}	Or7a-7	0.0022**		
	Or7a-1	0.0076**	0.1556$^{n.s.}$	
	Or7a-10	0.0091**	0.3843$^{n.s.}$	0.2501$^{n.s.}$
E(2)-hexenal 10^{-3}	Or7a-7	0.1096$^{n.s.}$		
	Or7a-1	0.0004***	0.0014**	
	Or7a-10	0.0232*	0.215$^{n.s.}$	0.0232*
E(2)-hexenal 10^{-4}	Or7a-7	0.7949$^{n.s.}$		
	Or7a-1	0.0008***	0.0004***	
	Or7a-10	0.0091**	0.0028**	0.1868$^{n.s.}$
E(2)-hexenal 10^{-5}	Or7a-7	0.4965$^{n.s.}$		
	Or7a-1	0.1971$^{n.s.}$	0.0751$^{n.s.}$	
	Or7a-10	0.0232*	0.0114*	0.7339$^{n.s.}$

Mann-Whitney *U*-test, 2-tailed.
